# Accelerated Aging Effect on Volatile Organic Compound Emissions from Thermally Treated Spruce Wood

**DOI:** 10.3390/ma19061135

**Published:** 2026-03-14

**Authors:** Tatiana Bubeníková, František Kačík, Anna Darabošová, Iveta Čabalová

**Affiliations:** Department of Chemistry and Chemical Technologies, Faculty of Wood Sciences and Technology, Technical University in Zvolen, T. G. Masaryka 24, 960 53 Zvolen, Slovakia; bubenikova@tuzvo.sk (T.B.); kacik@tuzvo.sk (F.K.); xdarabosova@is.tuzvo.sk (A.D.)

**Keywords:** thermal modification, volatile organic compounds, accelerated aging, Norway spruce (*Picea abies*), indoor air quality

## Abstract

Thermal modification is widely applied to improve the durability and dimensional stability of wood; however, it alters the emission profile of volatile organic compounds (VOCs), which may affect indoor air quality. This study evaluated the effect of accelerated aging on VOC emissions from thermally modified Norway spruce (*Picea abies*) wood. Untreated and thermally treated samples (160, 180, and 210 °C) were subjected to accelerated aging in a xenon test chamber for 600 h. VOC emissions were analyzed using headspace gas chromatography–mass spectrometry (HS-GC-MS), and total VOC emissions (TVOC) were calculated from peak areas. Thermal modification significantly reduced TVOC compared to untreated wood, with samples treated at 210 °C showing up to a 376-fold decrease. Increasing modification temperature reduced the amount and variability of emitted VOCs and altered their chemical composition. Terpenes dominated in untreated wood, particularly α-pinene (51%), whereas thermally treated samples showed lower terpene content and higher proportions of carbonyl compounds such as furfural. Accelerated aging further affected VOC emissions, including a 42% decrease in TVOC for the 160 °C sample and compositional shifts characterized by the disappearance or formation of specific compounds. Thermal modification and subsequent aging substantially modify VOC emission profiles and improve emission stability of thermally treated spruce wood.

## 1. Introduction

Global climate change requires a comprehensive approach to reducing greenhouse gas emissions and atmospheric carbon dioxide. Wood plays an important role in carbon storage, as plants absorb carbon dioxide during photosynthesis and incorporate it into biomass. Therefore, it is important to maintain carbon bound in wood for as long as possible. Various modification processes are used for this purpose to improve wood properties and extend its service life.

Among commercially used wood modification methods, thermal treatment occupies a significant position. Thermal processing of wood is primarily used to improve its dimensional stability under varying moisture conditions [1]. It represents a well-established commercial technology that enhances the appearance, dimensional stability, and durability of wood. Compared to other modification methods, its advantage is the absence of additional chemicals that could negatively affect the environment or the ecological quality of wood products [2,3,4]. Thermally modified wood is wood in which the composition of the cell wall material and its physical properties are altered by exposure to temperatures ranging from 160 °C to 260 °C [5] under conditions of reduced oxygen availability. The temperature and duration of thermal treatment typically range from 180 to 280 °C and from 15 min to 24 h, depending on the treatment process, wood species, sample dimensions, moisture content, and required mechanical properties, biological resistance, and dimensional stability of the final product [5,6,7]. This technology allows improvement of properties of lower-quality wood, which is subsequently used mainly as biomass for energy production or for packaging materials [8,9,10].

Despite these advantages, attention must be paid to the formation of substances generated during thermal modification that may have negative environmental or health impacts. Volatile organic compounds (VOCs) released from wood during the drying process have been extensively studied, particularly concerning the influence of temperature and moisture [11,12,13,14]. Currently, increasing attention is focused on VOC emissions from thermally modified wood. As these materials are increasingly used in indoor environments, it is necessary to evaluate their safety and impact on indoor air quality. This issue is particularly important because potentially toxic derivatives of polycyclic aromatic hydrocarbons (PAHs) have been identified in soluble extracts of thermally modified maritime pine and poplar wood [7]. VOC emissions from thermally modified wood have therefore raised concerns regarding indoor air quality and environmental impacts. Since people spend more than 80% of their time indoors, indoor air pollution caused by VOC emissions from wood and wood-based materials has been associated with the occurrence of sick building syndrome (SBS) [15,16].

The amount and composition of VOCs emitted from wood and wood-based materials depend on several factors, particularly wood species, treatment technology, and service history of the material. For example, hardwood species such as beech and oak primarily emit higher amounts of acetic and formic acids and lower amounts of terpene compounds, whereas low-density species such as poplar release lower amounts of organic acids but significantly higher amounts of terpenes [17]. VOC composition also differs depending on the type of wood-based composite material. The main components of VOCs emitted from particleboard include terpenes, alkanes, alcohols, aldehydes, esters, and aromatic compounds, while ketones occur in low concentrations. In fiberboards, terpenes, alcohols, and aromatic compounds dominate, whereas alkanes, aldehydes, ketones, and esters are present in lower amounts. In plywood, the major VOC components include terpenes, alcohols, ketones, esters, and alkanes, while aldehydes are present in low concentrations [15,18].

Thermal treatment significantly alters the chemical composition of wood, which is reflected in the profile of emitted VOCs. During thermal treatment of oak wood in an air atmosphere, eight groups of VOCs were identified, namely acids (37.05–42.77%), aldehydes (11.67–18.99%), ketones (11.49–18.94%), phenols (9.6–15.56%), furans (11.54–16.67%), alcohols (3.09–5.2%), sugars (1.53–3.22%), and esters (1.25–2.16%) [19]. In addition to temperature, the presence of inorganic constituents in wood may also influence thermal degradation processes. Wood naturally contains small amounts of mineral elements such as potassium, calcium, magnesium, and sodium, which remain in the ash fraction after thermal decomposition. These inorganic components can significantly affect the pathways of thermochemical reactions by acting as catalysts or inhibitors during biomass degradation. For example, alkali and alkaline earth metals can affect thermal decomposition kinetics and shift degradation reactions to lower temperatures, thereby influencing the distribution of volatile and solid products. Potassium, in turn, promotes the formation of low-molecular-weight compounds and suppresses the formation of levoglucosan during the thermal degradation of lignocellulosic biomass [20]. In addition, sodium and potassium are known catalysts in biomass thermal reactions and may significantly influence the rate of decomposition and the formation of degradation products [21]. More broadly, the presence of alkali and alkaline earth metals in biomass has been shown to influence the yields of char, gases, and volatile compounds during pyrolysis and other thermochemical conversion processes [22]. Surfaces of softwood species emit mainly terpenes, including mono-, di-, and sesquiterpenes, followed by aldehydes such as hexanal and pentanal [23]. In general, softwood species emit higher amounts of VOCs than hardwoods, mainly due to their higher content of volatile terpenes [24].

Thermal treatment processes alter the chemical composition of wood, leading to changes in VOC emission profiles. Accelerated aging may further influence emission characteristics and complicate the understanding of VOC release from thermally modified wood. Understanding changes in VOC emissions before and after aging is essential for assessing the environmental and health implications of thermally modified wood; however, studies addressing this issue remain limited.

This study aimed to evaluate the effect of accelerated aging on VOC emissions from thermally modified Norway spruce wood. The study focused on comparing the amount and composition of VOCs emitted from untreated and thermally modified wood at different modification temperatures, as well as evaluating changes in emission profiles after accelerated aging. It was hypothesized that thermal modification may lead to a significant reduction in total VOC emissions and simultaneously alter their chemical composition. Furthermore, it was assumed that accelerated aging affects both the quantity and spectrum of emitted compounds due to additional degradation and oxidation processes occurring in the wood matrix. It was expected that aging would reduce the amount of VOCs, thereby increasing the comfort of users of thermally modified wood. The results of this study may contribute to a better understanding of the behavior of thermally modified wood during its use in indoor environments.

## 2. Materials and Methods

### 2.1. Materials and Heat Treatment

**Figure 1:**
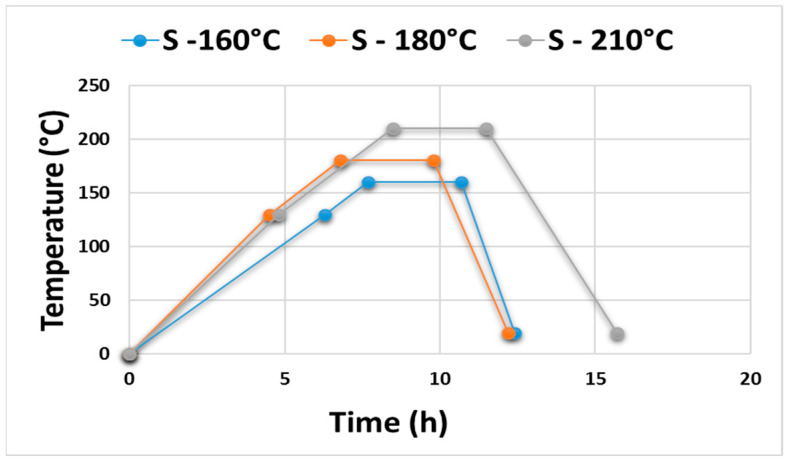
Temperature–time profile of the thermal modification process of Norway spruce wood according to Sikora et al. [25], including heating, modification, and cooling phases.

Volatile organic compounds (VOCs) were analyzed in untreated, thermally treated, and accelerated-aged Norway spruce (*Picea abies*) wood specimens with dimensions of 100 mm × 50 mm × 15 mm (length × width × thickness). The samples were divided into four groups, each containing 10 samples. One group served as a reference and was not subjected to thermal treatment. The remaining three groups were thermally modified using a laboratory thermal chamber S400/03 (LAC Ltd., Rajhrad, Czech Republic) according to the Thermowood process at temperatures of 160, 180, and 210 °C [25,26]. After reaching the desired temperature, the duration of the thermal treatment was three hours. The course of the thermal modification process is presented in Figure 1.

### 2.2. Accelerated Aging

Accelerated aging of the thermally modified wood samples was carried out in a xenon test chamber Q-SUN Xe-3-HS (Q-Lab Europe, Ltd., Bolton, UK) following the ASTM G155 standard (2005) [27] under wet conditions. Each group contained 5 samples. The total duration of the accelerated aging process was 600 h.

**Figure 2:**
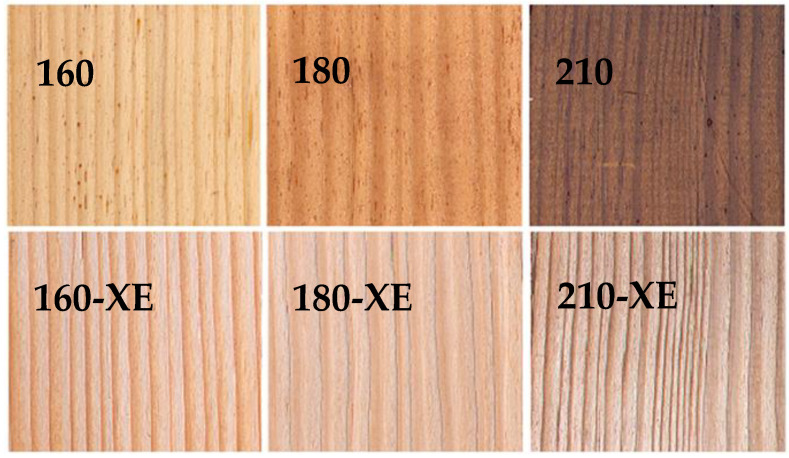
Visual appearance of thermally modified Norway spruce (*Picea abies*) wood samples at 160, 180, and 210 °C and thermally modified Norway spruce (*Picea abies*) wood samples at 160, 180, and 210 °C after accelerated aging (160-XE, 180-XE, 210-XE). Adapted from [28].

The designation of the test samples is presented in Table 1. Untreated spruce wood samples, serving as references, are labeled as 20. Samples thermally treated in a thermal chamber according to the Thermowood process at 160 °C, 180 °C, and 210 °C are labeled as 160, 180, and 210, respectively (Figure 2). Thermally treated samples subjected to accelerated aging are labeled as 160-XE, 180-XE, and 210-XE. Two replicates were prepared for each sample. This text provides additional clarification to Table 1, which summarizes all sample labels and their corresponding characteristics.

### 2.3. VOC Analysis

Spruce wood samples were ground into sawdust using a Polymix PX-MFC 90 D (Kinematica, Luzern, Switzerland) wood grinder at a rotational speed of 1200–1500 min^−1^. The sawdust was thoroughly mixed to ensure homogeneity and representativeness of the samples. Volatile organic compound (VOC) emissions were analyzed using the headspace gas chromatography–mass spectrometry (HS-GC-MS) technique. A 20 mL glass headspace vial was used as the VOC test chamber. Sawdust samples (0.500 ± 0.001 g) were placed into the vial and sealed immediately before analysis with an aluminum crimp cap equipped with a PTFE/silicone septum.

The headspace vial containing the sample was placed into the thermostat of the headspace autosampler. VOC analyses were performed using an Agilent 7890A gas chromatograph (Agilent Tehnologies, Santa Clara, CA, USA) coupled with a 5975C mass selective detector (MSD) (Agilent Tehnologies, Santa Clara, CA, USA) and equipped with an Agilent 7697A headspace autosampler (Agilent Tehnologies, Santa Clara, CA, USA). Two analytical replicates were performed for each sample.

The experimental conditions were as follows: Headspace conditions: carrier gas: helium; carrier gas pressure: 7.5 psi; oven temperature: 90 °C; loop temperature: 100 °C; transfer line temperature: 110 °C; vial equilibration time: 20 min.

GC and MS conditions: injection mode: headspace (180 °C), split ratio 20:1; column: HP-5MS (30 m × 0.250 mm × 0.25 μm); carrier gas: helium (constant flow rate 1.0 mL·min^−1^); oven temperature program: from 40 °C to 270 °C; transfer line temperature: 280 °C. MS temperatures were set to 230 °C for the ion source and 150 °C for the quadrupole. VOCs were identified by comparing the measured mass spectra with the NIST 20 mass spectral library. Peak areas were integrated using the MSD ChemStation 5.01 software and were used to compare the relative amounts of emitted VOCs. The sum of the areas of all detected peaks was calculated for each sample, and the relative percentage contribution of individual identified compounds was determined based on the total peak area. TVOC values are expressed as the sum of integrated peak areas in arbitrary units (a.u.).

## 3. Results and Discussion

**Figure 3:**
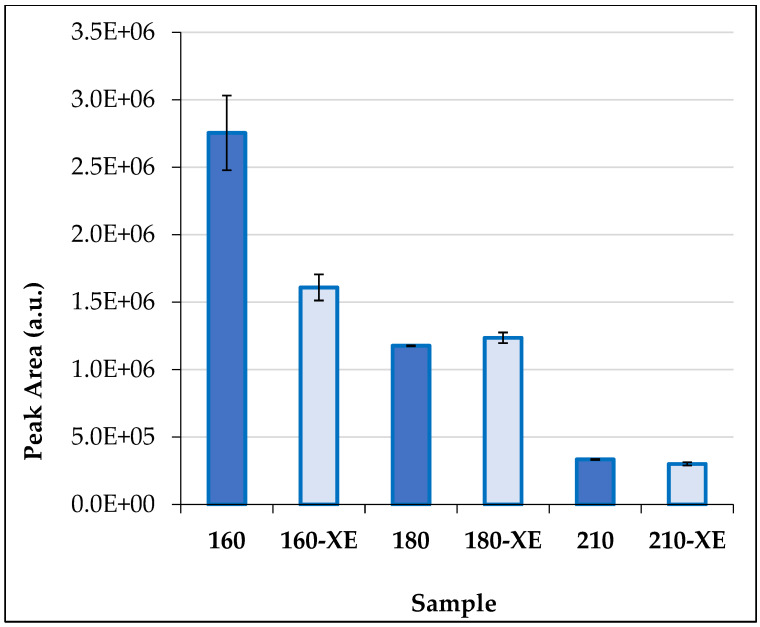
Total volatile organic compound emissions (TVOC) from thermally modified (160, 180, 210 °C) and accelerated-aged (160-XE, 180-XE, 210-XE) Norway spruce wood samples were determined by HS-GC-MS (mean ± standard deviation).

**Figure 4:**
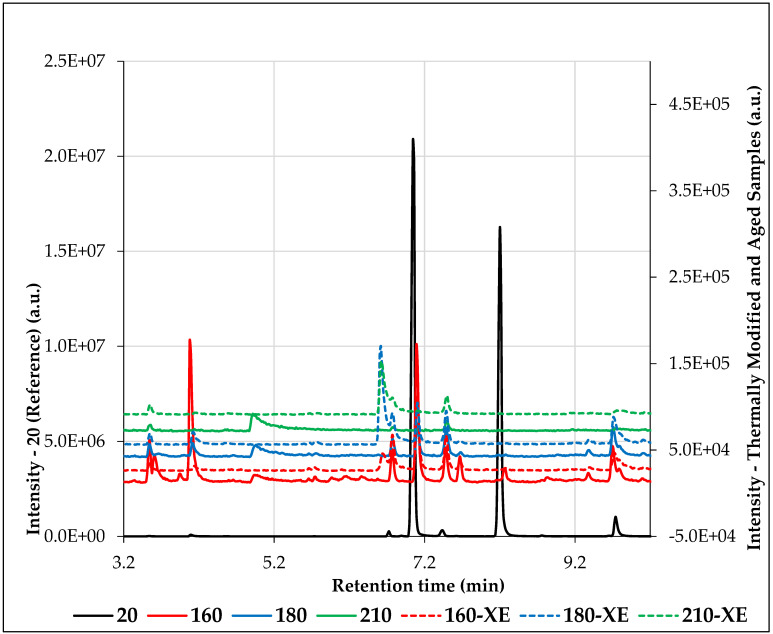
Representative HS-GC-MS chromatograms of VOC emissions from untreated (20), thermally modified (160, 180, 210 °C), and accelerated-aged (160-XE, 180-XE, 210-XE) Norway spruce wood samples.

A relatively large amount of volatile organic compounds (VOCs) was released into the air from unmodified spruce wood (reference sample—20). A total of 24 compounds were identified. The total peak area of emitted VOCs (TVOC) was significantly higher compared to all thermally modified samples (Table 2). With increasing thermal treatment temperature of spruce wood, a marked decrease in the amount of emitted VOCs was observed (Figure 3 and Figure 4), as well as a reduction in their variability (Table 2). TVOC released from spruce wood modified at 210 °C were up to 376 times lower compared to unmodified wood (Table 2).

These results are consistent with the work of Manninen et al. [29], who found that total VOC emissions from air-dried Scots pine wood blocks were seven- to nine-fold higher compared to emissions from thermally treated wood. Similarly, Hyttinen et al. [30], in a comparison of VOC emissions from several wood species during a four-week testing period, demonstrated that thermal treatment of spruce and pine wood significantly reduces total VOC emissions while simultaneously altering their chemical composition compared to untreated or naturally air-dried wood.

The dominant group of compounds in the VOC emission profile of the reference sample consisted of terpenes. The most significant volatile organic compound emitted from the reference sample was α-pinene, accounting for up to 51% of the total VOC content (Table 2). β-Pinene (39%) and β-terpinene (3%) were also present in substantial amounts. The monoterpenes α-pinene and β-pinene are considered the most important volatile organic compounds originating from coniferous species [31]. The dominant presence of α-pinene in VOC emissions from spruce wood was also confirmed by Raber et al. [32] in their study of emissions from blue spruce (*Picea pungens*) and Norway spruce (*Picea abies*) using the HS-GC-MS method. In addition to α-pinene and β-pinene, other VOCs typical of coniferous species were released from the reference sample, including caryophyllene, β-terpinene, bergamotene, camphene, and other compounds (Table 1).

Several studies indicate that terpenes may have negative effects on human health, particularly in indoor environments. They may cause irritation of the eyes, mucous membranes, and skin, trigger allergic reactions, and exacerbate respiratory problems, including asthma [33,34,35].

VOCs emitted into the atmosphere are chemically highly reactive and participate in various transformation processes, such as photolysis, reactions with hydroxyl (OH) and nitrate (NO_3_) radicals, reactions with ozone, and reactions with atomic chlorine [36]. A significant fraction of terpenes is oxidized by hydroxyl radicals. Based on the average global concentration of OH radicals, Montenegro et al. [37] estimated the atmospheric lifetime of α-pinene and β-pinene to be 5.8 and 3.8 h, respectively. Reactions of terpenes with ozone may lead to the formation of submicron particles, which significantly affect indoor air quality [38]. In the atmosphere, α-pinene reacts with oxidizing agents to form oxygenated and less volatile oxidation products [39].

VOCs are classified as primary and secondary. Primary VOCs are compounds naturally present in wood, whereas secondary VOCs are formed as a result of oxidative processes involving extractives and the cleavage of unsaturated fatty acids. Monoterpenes belong to primary VOCs, while aliphatic saturated aldehydes, such as hexanal and heptanal, are typical secondary VOCs. The formation of secondary VOCs depends on wood species, moisture content, storage temperature, and oxygen availability [40,41].

In addition to terpenes, hexanal, toluene, and bornyl acetate were identified in the emission profile of the reference sample. Carboxylic acids and aliphatic hydrocarbons were not identified in any of the samples using the applied analytical method.

Terpenes and carbonyl compounds, such as hexanal, furfural, 2-pinen-7-one (only in the sample modified at 160 °C), and toluene, were predominantly released from thermally modified spruce wood samples. In samples modified at 160 °C, the dominant VOCs were hexanal and α-pinene (Table 2). The increased proportion of hexanal at this temperature indicates a more intensive course of oxidative reactions and a higher concentration of free radicals in the system. The higher hexanal content may also result from an increased amount of free fatty acids, likely due to damage to the cellular structures of the wood. At the same time, several VOCs present in the reference sample, such as β-thujene, β-terpinene, β-myrcene, terpinolene, and bergamotene, were no longer identified in the thermally modified samples.

Although α-pinene remained among the dominant VOCs in thermally modified samples, its peak area in sample 160 reached only 0.74% of the α-pinene peak area of the reference sample. Prolonged exposure to high temperatures can significantly reduce terpene emission from wood [42]. The decrease in α-pinene content together with the presence of 2-pinen-7-one indicates ongoing oxidative reactions and thermal degradation of terpenes. Thermal degradation of α-pinene was also described by Punsuvon [43], who observed its partial oxidation and isomerization when heated in the presence of air at temperatures of 90–130 °C. In contrast to α-pinene, the peak area of hexanal in sample 160 increased by 67% compared to the reference sample.

Hexanal was also identified in samples modified at 180 °C; however, it was no longer among the dominant VOCs. Its peak area reached only 22% of the reference value. The dominant compounds at this temperature were p-cymene and furfural. Furfural was not identified in the reference sample, and its proportion increased with increasing thermal treatment temperature.

In samples modified at 210 °C, only three compounds were identified among the VOCs: toluene, furfural, and camphene. Increased furfural emissions may be attributed to hemicellulose degradation [31,44]. Thermal modification of wood is based on degradation and molecular restructuring of the cell wall, particularly of low-molecular-weight hemicelluloses [45]. The increased formation of furfural at elevated temperatures may also be explained by autohydrolysis reactions occurring within the wood structure. During heating in the presence of moisture, acetyl groups released from hemicelluloses generate hydronium ions, which catalyze the hydrolysis of hemicellulosic polymers. The liberated pentoses, particularly xylose, subsequently undergo dehydration to form furfural at elevated temperatures [46,47,48]. Sikora et al. [25] reported that modifying spruce wood at 210 °C led to a decrease in hemicellulose content of up to 10.85%.

Following thermal modification, vanillin, guaiacylacetone, and anethole were identified in pine species [49,50], while dodecane, tetradecane, and hexadecane were identified in Norway spruce [51]. Thus, thermal treatment significantly reduces VOC emissions and alters their composition, as also confirmed by [20].

Most materials exhibit decreasing VOC emission profiles over time [52,53]. The effect of aging on VOC emissions was also confirmed in this study. After accelerated aging (xenotest), differences were observed not only in the quantity but also in the composition of emitted VOCs. In the sample modified at 160 °C, TVOC decreased by 42% after xenotesting. In contrast, sample 180-XE showed a slight increase in TVOC, while in sample 210-XE the difference between TVOC values before and after xenotesting was approximately 10%.

Accelerated aging also affected the chemical composition of VOCs. The main aging factors affecting wood surfaces include ultraviolet radiation, moisture, temperature, and oxygen exposure, which trigger photochemical and oxidative degradation reactions [54,55,56,57]. Such processes lead to irreversible changes in the chemical structure of wood and accelerate surface degradation [58]. These mechanisms provide a plausible explanation for the altered VOC emission profiles detected after xenon-accelerated aging in the present study. After xenotesting, the aldehyde furfural was no longer identified among the emitted compounds. The disappearance of furfural after xenon aging may indicate its further participation in secondary oxidation or condensation reactions under UV exposure. β-Pinene was also not identified among the VOCs emitted from sample 160-XE. In contrast, the dominant compound in sample 180-XE was tricyclene, and in sample 210-XE it was longifolene, which had not been identified in the original sample modified at 210 °C. Tricyclene, a tricyclic monoterpene, naturally occurs in the essential oils of many plant species [59,60,61]. It may also be formed as a secondary product of terpene photooxidation, particularly during the photochemical transformation of monoterpenes such as α-pinene, β-pinene, or δ-3-carene, which are typical of coniferous species, or through acid-catalyzed isomerization of α-pinene [62]. Toluene, an aromatic VOC, is formed during the degradation of lignin and other aromatic components of wood. Aromatic hydrocarbons are known products of lignin degradation, formed through cleavage of aryl-O-R linkages and subsequent deoxygenation reactions of lignin-derived phenolic compounds. Toluene, in particular, has been reported to originate from deoxygenation of methylguaiacol derivatives and dehydroxylation of methylphenols formed during lignin decomposition [63]. Photooxidation and accelerated aging likely contributed to the slight increase in toluene content among the VOCs emitted from samples 180-XE and 210-XE.

## 4. Conclusions

This study aimed to evaluate the effect of thermal modification and subsequent accelerated aging on volatile organic compound (VOC) emissions from Norway spruce (*Picea abies*) wood. The research focused on comparing the quantity and chemical composition of VOCs emitted from untreated wood and wood thermally modified at 160 °C, 180 °C, and 210 °C, as well as assessing changes in emission profiles after accelerated aging in a xenon test chamber.

The experimental results confirmed that thermal modification led to a significant reduction in total VOC emissions (TVOC) compared to untreated wood, with emissions progressively decreasing as the treatment temperature increased. The most pronounced effect was observed at 210 °C, where TVOC values were up to 376 times lower than those of the reference sample. Simultaneously, the variability of emitted compounds decreased, and substantial changes in chemical composition were observed. While the emission profile of untreated wood was dominated by terpenes, particularly α-pinene and β-pinene, thermally modified samples exhibited a markedly lower terpene content and a higher proportion of carbonyl compounds, such as furfural.

Accelerated aging further influenced both the quantity and spectrum of emitted VOCs. In some cases, additional reductions in TVOC were recorded, whereas in others, slight variations in emission intensity were observed, accompanied by modifications in chemical composition. The disappearance of certain compounds and the formation of new components after xenotesting confirm that degradation and oxidation processes continue within the wood matrix even after thermal modification.

The results confirmed the proposed hypothesis that thermal modification significantly reduces VOC emissions from spruce wood and improves emission stability, which is particularly relevant for indoor applications regarding indoor air quality. Accelerated aging further modified both the quantity and composition of emitted VOCs, confirming that degradation and oxidation processes continue within the wood matrix even after thermal treatment. Although aging led to additional reductions in total VOC emissions in some cases, it also caused compositional shifts characterized by the disappearance of certain compounds and the formation of new ones. These findings highlight that long-term aging effects should be considered when evaluating the emission behavior of thermally modified wood intended for indoor use.

From a practical perspective, the results indicate that thermal modification can serve as an effective approach to reduce VOC emissions from spruce wood intended for indoor applications. The findings suggest that higher modification temperatures significantly decrease total VOC emissions, which may help optimize thermal treatment parameters during the production of low-emission wood materials. In addition, the obtained emission data may provide useful input for quality control procedures and for supporting environmental product declarations or other emission evaluation frameworks applied to wood-based products.

Future research should focus on the long-term emission behavior of thermally modified wood under real indoor environmental conditions, including the influence of humidity, temperature fluctuations, and natural light exposure. Further studies could also investigate the effect of different thermal treatment durations and regimes on VOC formation and stability. In addition, expanding the research to other wood species would provide a broader understanding of VOC emission mechanisms and support the development of practical guidelines for optimizing thermal treatment parameters to minimize VOC emissions.

## Figures and Tables

**Table 1:** Sample information.

Designation	Sample Characteristics
20	Reference—untreated spruce wood
160	Thermally treated in a thermal chamber at 160 °C
180	Thermally treated in a thermal chamber at 180 °C
210	Thermally treated in a thermal chamber at 210 °C
160-XE	Thermally treated samples at 160 °C subjected to accelerated aging
180-XE	Thermally treated samples at 180 °C subjected to accelerated aging
210-XE	Thermally treated samples at 210 °C subjected to accelerated aging

**Table 2:** Retention times and relative percentage contribution of individual identified volatile organic compounds (VOC) (determined based on the total peak area) released from untreated and thermally modified spruce wood samples.

RT (min)	VOC	20 (%)	160 (%)	180 (%)	210 (%)	160-XE (%)	180-XE (%)	210-XE (%)
3.55	Toluene	0.04	4.56	3.85	7.47	2.11	3.79	13.02
4.09	Hexanal	0.26	20.31	6.33	-	1.99	6.88	-
4.95	Furfural	-	3.35	12.84	65.9	-	-	-
6.73	Tricyclene	0.46	3.95	5.41	-	12.38	17.37	-
6.89	β-Thujene	0.07	-	-	-	-	-	-
7.05	α-Pinene	51.11	17.31	10.71	-	21.42	14.33	-
7.44	Camphene	0.96	5.11	10.23	12.03	10.86	12.82	26.06
8.2	β-Pinene	39.32	2.5	-	-	-	-	-
8.77	β-Myrcene	0.12	-	-	-	-	-	-
9.65	p-Cymene	0.05	5.78	16.97	-	11.33	16.13	-
9.74	β-Terpinene	3.02	-	-	-	-	-	-
11.54	Terpinolene	0.06	-	-	-	-	-	-
15.02	2-Pinen-7-one	-	4.8	-	-	8.36	-	-
17.11	Acetic acid, bornyl ester	0.21	-	-	-	-	-	-
18.75	α-Longipinene	0.08	-	-	-	-	-	-
20.12	Longifolene	0.71	4.45	3.12	-	4.09	2.69	37.85
20.3	α-Cedrene	0.19	-	-	-	-	-	-
20.5	Caryophyllene	0.54	-	-	-	-	-	-
20.91	α-Bergamotene	0.59	-	-	-	-	-	-
21.35	Humulene	0.06	-	-	-	-	-	-
21.92	Germacrene D	0.05	-	-	-	-	-	-
22.12	Curcumene	0.25	-	-	-	-	-	-
22.5	α-Muurolene	0.07	-	-	-	-	-	-
22.82	γ-Muurolene	0.26	-	-	-	-	-	-
23.04	δ-Cadinene	0.36	-	-	-	-	-	-
**TVOC (a.u.)**		125,971,841	2,754,337	1,176,745	334,360	1,608,750	1,235,620	300,651
**Identified Compounds (%)**		98.84	72.1	69.46	85.4	72.53	74.01	76.94

## Data Availability

The original contributions presented in this study are included in the article. Further inquiries can be directed to the corresponding author.

## Abbreviations

The following abbreviations are used in this manuscript:

VOCs	Volatile Organic Compounds
TVOC	Total Volatile Organic Compounds
HS-GC-MS	Headspace-Gas Chromatography–Mass Spectrometry
MSD	Mass Selective Detector
SBS	Sick Building Syndrome
PAH	Polycyclic Aromatic Hydrocarbons
UV	Ultraviolet (radiation)
ASTM	American Society for Testing and Materials
RT	Retention Time
a.u.	Arbitrary Units
